# Assisted Reproductive Treatments, Quality of Life, and Alexithymia in Couples

**DOI:** 10.3390/healthcare11071026

**Published:** 2023-04-04

**Authors:** Alessia Renzi, Fabiola Fedele, Michela Di Trani

**Affiliations:** 1 Department of Dynamic and Clinical Psychology and Health Studies, Sapienza University of Rome, Via Degli Apuli 1, 00185 Rome, Italy; 2 ART Italian National Register, National Centre for Diseases Prevention and Health Promotion, Italian National Health Institute, Viale Regina Elena 299, 00161 Rome, Italy

**Keywords:** alexithymia, quality of life, couple relationship, infertility, assisted reproductive treatment

## Abstract

Infertility and related treatments can negatively affect a couple’s wellbeing. The aim of this study was to evaluate couples starting assisted reproductive treatment, differences in alexithymia and quality of life levels between partners, and the association of these psychological dimensions within the couple’s members. Data was collected in two fertility centres in Rome; 47 couples completed the Fertility Quality of Life (FertiQoL), the 20-item Toronto Alexithymia Scale (TAS-20), and a socio-demographic questionnaire. Data analysis showed a worsened quality of life in women compared with their partners, as well as higher externally oriented thinking in men compared with their spouses. Associations between alexithymia and quality of life levels between women and men emerged. According to the regression analysis, a better quality of life in women was predicted by a greater partner’s capabilities in identifying and describing emotion as well as by a better partner’s quality of life, whereas for men, a better quality of life was predicted by their spouse’s higher levels of quality of life. This study highlights the protective role that couples can play in the perception of the negative impact that infertility can have on their partner’s quality of life. Further investigations are needed for the development of specific therapeutic interventions for the promotion of the couples’ wellbeing.

## 1. Introduction

Infertility is defined as the failure to achieve a clinical pregnancy after a year or more of regular, unprotected sexual intercourse [1]. According to this definition, the WHO estimated that in developed countries infertility is diagnosed in about 20% of reproductive-age couples. Specifically, in Italy it is estimated that an infertility diagnosis involves about 15% of couples [2]. Infertility can represent a major life crisis for couples since it threatens their life purpose related to parenthood. Both the infertility diagnosis and its associated treatments can be accompanied by a variety of psychological disorders [3,4]. Several studies reported that infertile couples tend to experience a varied range of negative emotions, including anxiety, fear, avoidance, depression, guilt, and frustration, all of which can impact overall life satisfaction and well-being [5,6,7,8,9,10,11,12].

The construct of quality of life is defined as “an individual’s perception of their position in life in the context of the culture and value systems in which they live and in relation to their goals, expectations, values, and concerns incorporating physical health, psychological state, level of independence, social relations, personal beliefs, and their relationship to salient features of the environment. Quality of life refers to a subjective evaluation that is embedded in a cultural, social, and environmental context” [13]. International literature showed that infertility strongly impacts the quality of life of people facing this condition, thereby impairing the physical, emotional, social, relational, and sexual dimensions, with women being more negatively affected than their partners [14,15,16,17,18,19,20,21].

Therefore, childlessness and the stigma associated with infertility conditions and treatments have the potential to seriously stress a couple’s relationship, possibly destroy their marriage or strengthen the bond between the partners [22,23,24,25,26]. According to previous studies, the positive or negative effect that infertility can have on a couple’s relationship seems to depend on the communication and support that the partners are able to provide to each other [27]. In fact, open communication between both partners can have a protective role in overcoming challenging experiences in the marital relationship [28]. Therefore, couples facing fertility problems who can openly share their stresses and pains can provide each other with support during this difficult journey so that infertility can possibly even bring the couple closer together and strengthen their intimate relationship, thereby increasing the feeling of cohesion within the couple [28,29,30,31].

In this regard, the alexithymia construct can play an important role. Alexithymia is a multidimensional construct characterised by difficulties in identifying feelings and in finding words to describe feelings to others, constricted imaginal processes, and an externally oriented cognitive style [32]. More recently, it has been conceptualised as an emotion-dysregulation disorder, including difficulties in using fantasy to regulate painful feelings and in finding creative solutions to problems or communicating their needs to others to obtain social support [33]. Moreover, the construct has been associated with difficulties in romantic relationships since engaging in intimacy may be difficult for individuals with scarce abilities to disclose emotional and personal information and empathic competencies [34]. In this regard, little research has been conducted on alexithymia in couples, showing its negative association with marital adjustment [35,36,37,38]. Moreover, alexithymia was related to the use of dysfunctional coping strategies [39,40,41,42], which makes it conceivable to consider people with higher levels of alexithymia as facing greater difficulties in coping with stressful conditions such as infertility and/or related medical procedures [41].

Across international literature, there is a relative paucity of studies exploring the role of alexithymia in infertility in couples since the investigation on this theme is mainly focused on the comparison of infertile and fertile individuals, constantly finding higher levels of alexithymia in people suffering from fertility problems [43,44,45,46]. Therefore, it seems important to specify the substantial need for intra-couple studies in addition to inter-couple studies. The investigation of the association between alexithymia and other psychological dimensions or symptoms showed positive relations between alexithymia and anxiety [46,47], depression, physical problems [47], emotional maladjustment to infertility [48], and maladaptive coping strategies and stress [41]. Furthermore, alexithymia was a significant predictor of infertility-related stress [49,50].

In conclusion, it seems crucial to have a good understanding of one’s emotions and needs when dealing with stressful conditions. Therefore, alexithymia may influence the quality of life of people facing infertility and related treatments, as both can directly impact a couple’s relationship.

Specific investigations of the association between alexithymia and quality of life in infertility, with a focus on couples, belong to a neglected area of research. Therefore, the aims of the present study were to evaluate the following in couples undergoing assisted reproductive technique (ART) treatments: (1) possible differences in alexithymia levels and quality of life, assessed from an inter-partner perspective; (2) the association between alexithymia and quality of life, assessed separately for women and men; (3) the association between alexithymia and quality of life, assessed from an inter-partner perspective; and (4) the predictors of a better quality of life, assessed separately for women and men. According to the international literature, we hypothesise that women will have a worsened quality of life and lower levels of alexithymia compared to their partners. Moreover, we hypothesise that a higher level of alexithymia will be associated with a worsened quality of life. We also hypothesise that higher alexithymia levels, indicating greater difficulties in affect regulation, will have a significant predictive effect on the quality of life in both intra- and inter-partner relationships.

## 2. Materials and Methods

### 2.1. Sampling and Recruitment

According to the Italian law (n. 40/2004) regulating the implementation of ART treatment in Italy, participants were heterosexual couples who were undergoing an ART treatment in two assisted reproduction centres in Rome according to the following inclusion criteria:

- Over 18 years of age;
- Undergoing an in vitro fertilisation treatment with embryo transfer (IVF-ET), intra-cytoplasmic sperm injection (ICSI), or intrauterine insemination (IUI);
- Childless couples;
- Having their first ART cycle medical visit at the centre;
- Undergoing ART treatment for a fertility problem and not for pre-implantation genetic diagnosis (PGD);
- Possessing an adequate understanding of the Italian language.

We excluded women with an inadequate understanding of the Italian language and/or those with a history of psychiatric disorders, defined as having received a psychiatric diagnosis and/or a pharmacological treatment for a psychiatric disorder during the course of their life.

During the screening for eligibility (see Section 2.3), 12 couples (18.75%) were excluded (n = 7 had children; n = 2 showed an inadequate understanding of the Italian language; n = 3 used ART for pre-implantation genetic diagnosis). Fifty-two eligible couples (81.25%) were invited to participate in the study; 47 (90.38%) agreed to participate while five (9.62%) declined. Non-participation was mainly due to time constraints and rarely to male reluctance.

A total of 47 couples (94 individuals) participated in the present study. All were married or cohabiting, with the average number of months passing since the beginning of their attempts at pregnancy being 16.22 (SD = 19.81). Regarding the infertility cause, 12.9% reported an unknown cause, 40.4% reported a female cause, 34.1% reported a male cause, and 12.8% reported a cause involving both partners. 59% of the couples pursued an in vitro fertilisation treatment with embryo transfer (IVF-ET) or an intra-cytoplasmic sperm injection (ICSI) treatment for fertility, and 76.6% were at their first ART attempt. The average age of women in the study was 35.98 years (SD = 4.87). Regarding levels of education for women, 19.1% completed 8 years of school, 40.4% completed 13 years of school, and 40.5% completed ≥ 16 years of school. Regarding occupation, 42.6% were public or private sector employees, 21.2% were homemakers, and 36.2% were unemployed. 10.6% of women reported a history of miscarriages. The average age of men in the study was 37.36 years (SD = 5.68). Regarding levels of education, 17% completed 8 years of school, 53.2% completed 13 years of school, and 29.8% completed ≥ 16 years of school. Regarding occupation, 42.6% were public or private sector employees, 8.5% worked freelance jobs, 29.8% identified as workers, and 2.1% were unemployed.

### 2.2. Measures

A socio-demographic questionnaire was designed to collect information on age, gender, social status, level of education, employment, cause of infertility, time since the beginning of pregnancy attempts, number of previous ART treatments, and history of psychiatric disorders.

The 20-item Toronto Alexithymia Scale (TAS-20) [51,52] is the most employed instrument for the assessment of alexithymia. It is structured according to three factors: Difficulty in Identifying Feelings (DIF; seven items), Difficulty in Describing Feelings (DDF; five items), and Externally Oriented Thinking (EOT; eight items). Each item is rated on a 5-point Likert scale ranging from (1) ‘strongly disagree’ to (5) ‘strongly agree’. This instrument provides both a total score and a score for each factor. Higher scores indicate higher levels of alexithymia. In the present study, Cronbach’s alpha for the total score was 0.83.

The Fertility Quality of Life (FertiQoL) [53,54] was used to measure the quality of life in people struggling with fertility. It is divided into two modules: I. The Core FertiQoL, consisting of 24 items exploring four subscales: (1) Emotional, exploring the impact of infertility on emotions (six items); (2) Mind-Body, evaluating the impact on physical health (e.g., pain and fatigue), cognition (e.g., attention), and behaviour (e.g., change in daily activities), based on the notion that attention and concentration are impaired by thoughts of infertility (six items); (3) Relational, evaluating the impact of the condition of infertility on partnerships (six items); and (4) Social, exploring the impact on social aspects (e.g., social inclusion and stigma), such as feeling uncomfortable attending social situations, such as holidays, because of fertility problems (six items); II. The Treatment FertiQoL is an optional module consisting of 10 items. In the present study, only the Core module has been used since the couples have not yet started the medical procedure, so evaluating the impact of treatment dimensions on their quality of life was not possible. Each item is scored according to five response categories, ranging from 0 to 4. Scaled scores ranged from 0 to 100, with higher scores on the total FertiQoL scale or any subscale referring to a better quality of life. The Italian version of the psychometric properties has been confirmed in previous investigations [17,55]. In the present study, total and subscale Cronbach’s alpha were deemed acceptable (being in the range of 0.76 and 0.91).

### 2.3. Procedure

The study was conducted between November 2021 and April 2022. The work had been carried out in accordance with the code of ethics of the World Medical Association (Declaration of Helsinki) for experiments involving humans. Ethical approval was granted by the Ethics Committee of our university department.

The couples were informed of the study during their visit to the centre, which preceded the beginning of the ART procedure. The gynaecologist and the psychologists responsible for the research protocol screened the couples for eligibility according to the information collected in the medical records. Further, the exclusion/inclusion criteria were routinely explored during the anamnestic process. After the medical visit, the gynaecologist introduced the consecutively admitted eligible couples to the psychologist responsible for the research protocol’s implementation. The psychologist invited the couples to contribute to a study “aimed to investigate how women and men feel about the infertility experience”. The couples who agreed to participate signed an informed consent form before completing the tests. The research protocol took place in the medical centres and was implemented by a qualified psychologist in a dedicated room.

### 2.4. Statistical Analysis

The statistical analyses were conducted using the Statistical Package for Social Science (SPSS) version 24 for Windows. Data were reported as frequencies and percentages for discrete variables, as well as means and standard deviations for continuous variables. *T*-tests for paired samples were used to evaluate possible differences between partners in alexithymia and quality-of-life scores. Cohen’s d has been calculated to detect a measure of the effect size. Pearson’s correlation was used to measure the association between levels of alexithymia, quality of life, and socio-medical variables among inter- and intra-groups of women and men, respectively. A set of multiple regression analyses were performed in order to investigate the role of alexithymia scores, both individually and with their partners (respectively, total and factor scores). Further, the socio-medical variables were investigated in the present study to explain the variance in the quality-of-life scores of women and men. More specifically, in the set of models tested, the Core FertiQoL scores of both the female and male partner, respectively, were used as dependent variables, whereas the variables age and alexithymia scores (respectively and alternately, TAS-20 total scores and factor scores), as well as the partners’ quality-of-life score, were used as independent variables. All the variables were entered simultaneously. A *p* < 0.05 was considered significant.

## 3. Results

Regarding differences between partners in alexithymia and quality of life, several significant differences emerged with a medium effect size regarding infertility-related quality of life dimensions and a small to medium effect size for alexithymia (Externally Oriented Thinking) (see Table 1).

Regarding correlation analyses between levels of alexithymia and quality of life within the group of women, only a significant relationship emerged between Difficulties in Identifying Feelings and the Emotional Dimension of FertiQoL (r = −0.308; *p* < 0.05). No associations between alexithymia and quality of life in men emerged. Some significant, moderate-to-strong associations between alexithymia and quality of life emerged (see Table 2).

A set of multiple linear regression analyses was performed using the Total Core FertiQoL of women and men, respectively, as dependent variables; age; levels of alexithymia for both partners (respectively and alternatively, total and factor scores); and the partner’s quality of life as independent variables. The first model explains 41.8% of the women’s Total Core FertiQoL (R^2^ = 0.42; adjusted R^2^ = 0.36). Further, the independent variable shows the only significant effect being the partners’ quality of life (β = 0.562; *p* < 0.001). In order to further investigate the role of alexithymia, the regression analysis was repeated using the TAS-20 factor scores as independent variables. This model explains 50% of the FertiQoL total scores (R^2^ = 0.50; adjusted R^2^ = 0.42). The independent variables showing significant effects were: partners’ quality of life (β = −0.573; *p* < 0.001), and partners’ Difficulties in Identifying Feelings (β = −0.378; *p* = 0.021) and Difficulties in Describing Feelings (β = −0.335; *p* = 0.049). Regarding men’s Total FertiQoL Core scores, the model explains 36.7% of the scores (R^2^ = 0.37; adjusted R^2^ = 0.30), and the only significant independent variable was women’s quality of life (β = 0.620; *p* < 0.001). The regression analysis was repeated using the TAS-20 factor scores as independent variables, and this model explains 42% of the scores (R^2^ = 0.42; adjusted R^2^ = 0.29) and the only significance of women’s quality of life was confirmed (β = 0.654; *p* < 0.001).

## 4. Discussion

We sought to examine intra-couple differences in quality of life and alexithymia scores between women and men, hypothesising that women would show a worse infertility-related quality of life and less difficulty with affect regulation than men. Our findings confirmed this study hypothesis since women scored significantly lower than their partners (Cohen’s d ranging from 0.637 to 0.439) on Emotional, Mind-Body, Social, and Total Core FertiQoL, thereby reporting a worse infertility-related quality of life. This finding appears to be coherent with the broader literature on the theme highlighting that women’s well-being and psycho-physical health are more negatively affected by the infertility diagnosis and treatments than men [23,53,56,57]. Regarding the aexithymia construct, men reported higher levels of Externally Oriented Thinking than their spouse (Cohen’s d = 0.373), therefore showing an externally oriented cognitive style. This result appears to be in line with previous findings that reported that men were more alexithymic than women [58], and specifically with those reporting that men were more externally oriented in their thinking than women, even though both partners did not differ in their ability to identify feelings [59]. It seems possible to hypothesise that having the capability to identify and describe feelings to others and displaying a greater disposition to externally oriented thinking may reduce the negative impact of stressful conditions, such as an infertility diagnosis, with men acting as a defensive protective factor.

Regarding the investigation of the associations between alexithymia and quality of life in infertile women and men, separately, only a significant and negative relation emerged in women between Emotional dimensions of Quality of Life and Difficulties in Identifying Feelings. Thus, in women, higher levels of this alexithymic characteristic appeared to be associated with a greater negative impact of infertility on emotions (e.g., causing more negative feelings such as sadness, resentment, and grief). A general difficulty in accessing emotions makes it more difficult to process the specific effects related to the experience of infertility. No significant associations emerged within the group of men. This result only marginally confirmed our hypothesis for women and surprisingly seemed to support the absence of an association between emotional capabilities and quality of life. This appeared to be confirmed when also exploring the associations between women’s levels of alexithymia and men’s levels of quality of life, and vice versa, since no significance emerged. These findings appeared to be in contrast with the broader literature on this argument, both in regard to infertility and to other psychophysical conditions [41,50,60,61].

Several considerations can be made. The first being a comparison between the mean scores of alexithymia and quality of life obtained by the participants of this study and those of other investigations. More specifically, it can be qualitatively observed that women and men showed a TAS-20 mean total score similar to the mean scores obtained by the general Italian population (m = 44.7; SD = 11.3) [52]. Therefore, this data does not seem to highlight great difficulties in affect regulation, consistent with what has been shown by Gourounti et al. [41] (m = 49.07; SD = 11.9), Lamas et al. (2006) [44] (m = 47; SD = 11.8), and Moreno-Rosset et al. [45] (m = 46.49; SD = 13.16). On the contrary, women’s mean scores of infertility-related qualities of life are higher than those reported by women in the validation of the instrument (m = 53.3; SD = 16.2) [53], whereas men’s mean scores are similar to those found in the validation article (m = 72.1; SD = 14.7). It is possible to hypothesise that the sample recruited in the present study, which was characterised in 76.6% of cases by people at their first attempt, has not yet been impacted by the challenges of the medical procedure. It is also possible that the timing of the study, during the first visits to the centre, emphasises a valuing dimension, inducing women to lessen the impact of infertility on their quality of life in order to give more socially desirable answers. All of this may have produced a bias in potentially explaining the absence of associations between alexithymia and quality of life, especially in women.

Some significant associations between couples emerged for both levels of alexithymia and quality-of-life scores, separately for each construct. In fact, men’s Difficulties in Identifying Feelings showed positive associations with women’s Difficulties in Describing Feelings, Externally Oriented Thinking, and total scores of alexithymia. Moreover, women’s Emotional, Mind-Body, Social, and Total Core quality of life dimensions were positively associated with the same dimensions in men, with all associations ranging from moderate to strong. Women’s Relational scale showed a positive correlation only with men’s Social and Total scores. Within a systemic and circular perspective, it is possible to consider that one partner’s functioning can be affected by the other partner’s characteristics, as widely recognised in several contexts of investigations (e.g., trauma, cancer, etc.) [62,63]. Thus, the present findings seem to support the hypothesis of a systemic influence on quality of life within the couple, with a partner’s better quality of life being associated with a better personal quality of life.

The regression model tested the overall level of women’s infertility-related quality of life as predicted by their partners’ total quality of life and difficulties in identifying and describing emotional scores. These results appeared to be in line with those in [64], showing that men’s infertility-related distress predicted women’s psychological distress. On the contrary, in men, the quality-of-life result was predicted only by their spouse’s quality of life, and no significant roles of alexithymia (both individual and their partners’) emerged. In our opinion, these findings are interesting and support the importance of a partner’s wellbeing for an individual’s own sense of well-being when facing a difficult condition such as infertility. Moreover, the protective role of partners’ good capabilities in emotion identification and description appeared to be confirmed for women. It is possible that in the infertility framework, the possibility of sharing the difficult experience with a partner, as well as the ability to self-disclose, empathise with, and be affectively attuned to their partner, can represent protective factors for women living this challenging experience [31].

### Limitations and Strengths

These findings need to be interpreted in light of some limitations. First, the limited sample size can reduce the generalizability of findings; thus, studies with more participants should be completed. Furthermore, the sample was drawn from just two infertility centres in Rome, and this may have introduced a selection bias. Moreover, a potential social acceptability bias should be considered regarding the impact of infertility on the quality of life. In this light, the use of self-report instruments appeared as a limitation, and future studies should aim to include a clinical interview or clinician-report instrument. Finally, the absence of a control group is a further limitation that should be overcome in future investigations.

Despite these limitations, this study is, to our knowledge, one of the few studies that investigated the alexithymia construct in association with the quality of life in people struggling with infertility, with a specific focus on the couple dimension. Future studies should aim to employ specific data analysis methods for dyads/couples, such as the Actor-Partner Interdependence Model [65].

## 5. Conclusions

The study supports a worsened quality of life in women compared to their partners, and the protective role that the couple can play in the perception of the negative impact of infertility on both partners’ quality of life. In fact, partners’ good quality of life predicted a better quality of life in both the couple’s members. Moreover, for women’s quality of life, their partners’ emotional capabilities play an important role. A better understanding of the couple’s experience of infertility and related treatment may be crucial to developing and promoting intervention programmes aimed to enhance communication and mutual disclosure between the couples, as well as mutual support between partners. This finding may have also relevant implications for ART clinical staff since better wellbeing, lower psychological symptoms, and greater emotion regulation capabilities may improve centres’ success rates due to the positive association between quality of life and positive ART outcomes [66,67,68,69,70].

In future studies, it may be interesting to further explore couples’ dynamics, couples’ relationship satisfaction, perceived support from their partner, communication style, and dyadic coping strategies.

## Figures and Tables

**Table 1:** Differences in Fertility Quality of Life and alexithymia between the couple’s members.

	Women		Men		t	*p*	Cohen’s d
	M	SD	M	SD			
FertiQoL Emotional	58.33	22.4	72.03	16.87	−3.946	0.001	0.558
FertiQoL Mind-Body	60.93	23.07	72.96	20.09	−4.228	0.001	0.630
FertiQoL Relational	79.40	14.09	82.28	12.64	−1.200	0.237	0.178
FertiQoL Social	64.22	20.75	71.87	16.68	−2.949	0.049	0.439
Total Core FertiQoL	65.74	16.97	74.79	13.75	−4.278	0.000	0.637
TAS-20 DIF	14.70	6.74	13.63	6.16	0.876	0.386	0.001
TAS-20 DDF	12.20	4.06	11.76	4.43	0.573	0.570	0.080
TAS-20 EOT	17.15	3.86	19.22	4.31	−2.540	0.015	0.373
TAS-20 Total	44.04	11.15	44.61	12.35	−0.263	0.794	0.038

Legend. FertiQoL = Fertility Quality of Life; TAS-20 = 20-item Toronto Alexithymia Scale; DIF = Difficulty in Identifying Feelings; DDF = Difficulty in Describing Feelings; EOT = Externally Oriented Thinking.

**Table 2:** Association between alexithymia and quality of life in women and men.

	Women									
**Men**		**TAS-20** **DIF**	**TAS-20 DDF**	**TAS-20 EOT**	**TAS-20 Total**	**FeriQoL Emotional**	**FertiQoL Mind-Body**	**FertiQoL** **Relational**	**FertiQoL** **Social**	**Total** **Core** **FertiQoL**
	TAS-20 Total	0.095	0.281	0.206	0.231	0.140	0.115	−0.088	0.093	0.095
	TAS-20 DIF	0.222	0.291 *	0.329 *	0.331 *	0.116	0.122	0.031	0.102	0.118
	TAS-20 DDF	−0.012	0.266	0.027	0.099	0.041	0.003	−0.141	−0.007	−0.019
	TAS-20 EOT	0.022	0.116	0.091	0.087	0.193	0.153	−0.151	0.129	0.122
	FertiQoL Emotional	−0.200	0.065	0.021	−0.092	0.323 *	0.431 **	0.251	0.492 **	0.450 **
	FertiQoL Mind-Body	−0.044	0.128	0.002	−0.346 **	0.512 **	0.616 **	0.272	0.543 **	0.599 **
	FertiQoL Relational	0.035	0.072	0.024	0.056	0.126	0.211	0.278	0.224	0.237
	FertiQoL Social	−0.062	0.173	−0.139	−0.024	0.444 **	0.530 **	0.426 **	0.587	0.593 **
	Total Core FertiQoL	−0.126	0.136	−0.030	−0.038	0.451 **	0.567 **	0.370 *	0.579 **	0.591 **

* p < 0.05; ** *p* < 0.01. Legend. FertiQoL = Fertility Quality of Life; TAS-20 = 20-item Toronto Alexithymia Scale; DIF = Difficulty in Identifying Feelings; DDF = Difficulty in Describing Feelings; EOT = Externally Oriented Thinking.

## Data Availability

The data that support the findings of this study are available upon request from the corresponding author.
